# Inoculation route-dependent Lassa virus dissemination and shedding dynamics in the natural reservoir – *Mastomys natalensis*

**DOI:** 10.1080/22221751.2021.2008773

**Published:** 2021-12-06

**Authors:** D. M. Wozniak, S. A. Riesle-Sbarbaro, N. Kirchoff, K. Hansen-Kant, A. Wahlbrink, A. Stern, A. Lander, K. Hartmann, S. Krasemann, A. Kurth, J. Prescott

**Affiliations:** a ZBS5—Biosafety Level-4 Laboratory, Robert Koch-Institute, Berlin, Germany; b Institute of Neuropathology, University Medical Center Hamburg-Eppendorf, Hamburg, Germany

**Keywords:** Lassa virus, LASV, *Mastomys natalensis*, dissemination, shedding, natural reservoir, zoonosis, Lassa fever

## Abstract

Lassa virus (LASV), a Risk Group-4 zoonotic haemorrhagic fever virus, affects sub-Saharan African countries. Lassa fever, caused by LASV, results in thousands of annual deaths. Although decades have elapsed since the identification of the Natal multimammate mouse (*Mastomys natalensis*) as a natural reservoir of LASV, little effort has been made to characterize LASV infection in its reservoir. The natural route of infection and transmission of LASV within *M. natalensis* remains unknown, and the clinical impact of LASV in *M. natalensis* is mostly undescribed. Herein, using an outbred colony of *M. natalensis*, we investigate the replication and dissemination dynamics of LASV in this reservoir following various inoculation routes. Inoculation with LASV, regardless of route, resulted in a systemic infection and accumulation of abundant LASV-RNA in many tissues. LASV infection in the Natal multimammate mice was subclinical, however, clinical chemistry values were transiently altered and immune infiltrates were observed histologically in lungs, spleens and livers, indicating a minor disease with coordinated immune responses are elicited, controlling infection. Intranasal infection resulted in unique virus tissue dissemination dynamics and heightened LASV shedding, compared to subcutaneous inoculation. Our study provides important insights into LASV infection in its natural reservoir using a contemporary infection system, demonstrating that specific inoculation routes result in disparate dissemination outcomes, suggesting intranasal inoculation is important in the maintenance of LASV in the natural reservoir, and emphasizes that selection of the appropriate inoculation route is necessary to examine aspects of viral replication, transmission and responses to zoonotic viruses in their natural reservoirs.

## Introduction

Lassa fever (LF) is an often fatal haemorrhagic fever in humans, and Lassa virus (LASV) was identified as the etiologic agent of LF over 50 years ago [1,2]. LF is fatal in approximately 20% of diagnosed cases [3] and no clinically approved therapeutics or vaccines exist to counter the disease. The symptoms of LF are multifarious and can present with fever, abdominal pain, diarrhoea, headache, cough, malaise, facial oedema, oral ulcers, petechia and haemorrhage, as well as neurological complications and multiorgan failure [4]. Older estimations approximated 5,000 deaths and 300,000–500,000 infections with LASV in western Africa, annually [5]. Due to increased human population densities in western Africa since these estimations, the current LASV infection toll is likely far higher, deduced by increasing incidences of LF in recent years in Nigeria [3]. LF, therefore, cumulatively kills more people than any other viral haemorrhagic fever, excluding yellow fever [6] and severe dengue [7], for which vaccines exist. Within the often-underfunded healthcare systems of these economically less fortunate western African countries, which are now additionally struck by the SARS-CoV-2 pandemic, LF presents a great burden. Excluding western African countries, LASV also presents dangers to non-African countries by imported cases and secondary infections due to human-to-human-transmission, as has occurred repeatedly [8,9].

Natal multimammate mice (*Mastomys natalensis*), living peri-domestically and abundant throughout much of Sub-Saharan Africa, was elucidated as a LASV natural reservoir rapidly after LASV’s initial discovery [10], a feat achieved for other Risk Group-4 non-arboviruses only after decades of investigation [11–13]. Preliminary infection studies with Natal multimammate mice as a reservoir for LASV hinted at prolonged viral persistence of at least 103 days in adult Natal multimammate mice*,* and systemic chronic infections of up to 74 days in neonates [14]. The geographic ecology of LASV in *M. natalensis* has been researched more extensively, showing the spread of LASV within its host populations throughouts sub-Saharan western Africa. Virus detection and seroprevalences range between 10% and 30% in rodent populations, regionally, generally increasing with rodent age [5,10,15–18]. Human populations display regional seroprevalences between 10% and 52% [5,18–20]. A minority of LF cases stem from human-to-human transmissions in nosocomial settings, and though these outbreaks garner much attention [1,21], the vast majority of human infections are zoonotic, demonstrated by LASV genome sequencing during spill-over [22]. Transmission within *M. natalensis* populations likely occurs *in utero* and during perinatal contact, deduced from preliminary studies on *M. natalensis* and other arenaviruses in their natural reservoirs [14,23–26], however, infection of naïve adult animals and the virus dynamics in *M. natalensis* have not been sufficiently researched. Human spill-over is generally thought to occur by contamination of foodstuffs and surfaces with LASV-contaminated faeces or urine of *M. natalensis* [27] but has not been definitively determined.

Since almost nothing is known regarding the dynamics of LASV infection, tissue tropism, and the influence of inoculation route on infection outcome in the natural reservoir, for the first time, we used a stable non-inbred colony originating from wild Natal multimammate mice [28–30] to examine the clinical progression and temporal organ dissemination of LASV, resulting from disparate inoculation routes in adult Natal multimammate mice in a controlled laboratory setting. We analysed viral RNA burden throughout infection in all major organs and blood and examined oral, rectal and urinary shedding. Importantly, we examined the influence of the route of inoculation on dissemination and shedding of LASV*,* including intraperitoneal (i.p.) inoculation as a basis for a systemic infection, and compared intranasal (i.n.) and subcutaneous (s.c.) inoculation as potentially important for LASV maintenance within reservoirs. All three inoculation routes resulted in systemic infections and histologic inflammatory changes. However, i.n. inoculation of Natal multimammate mice uniquely resulted in detectible shedding of LASV-RNA, compared to s.c. inoculation, identifying i.n. as likely important for intraspecies transmission. Furthermore, i.n. inoculation of LASV resulted in a unique signature of LASV replication dynamics with increased lung tropism and higher overall viral burden, resulting in unique histological changes.

## Material and methods

### Animals

Male and female *M. natalensis* (8–16 weeks old), outbred descendants from wild-caught animals from Mali (phylogroup A-I) [28,29,31], were bred and maintained at the animal facility of the Robert Koch Institute (RKI), Berlin.

Experimental procedures conformed to German animal protection laws and the experimental protocol was approved by the responsible animal ethics committee (LaGeSo Berlin, Germany). All staff handling the animals were adequately trained and qualified on basis of specifications from Federation of European Laboratory Animal Science Associations. Euthanasia was performed by isoflurane overdose with exsanguination via cardiac puncture and cervical dislocation.

Animals were housed in IVC-Cages in groups of three littermates (Tecniplast Green line GR900), with dust-free woodchip bedding, tissue nesting material and red plastic tunnels at 22°C, 55% RH and a 12-hour photoperiod. Standard rodent chow and autoclaved tap water were provided *ad libitum*. The animals were acclimated to BSL4 conditions for at least 7 days prior to experimentation. Animals were anaesthetized with isoflurane for all handling in the BSL4 laboratory. Experimental groups were age-matched and sex-balanced as closely as possible, sourcing each cage of a group from different breeding pairs, thereby increasing the genetic heterogenicity.

### LASV and inoculation of natal multimammate mice

The human-isolate Lassa virus (LASV) strain, Josiah (GenBank Accession: NC_004296.1, NC_004297.1) [32], was propagated on Vero76 cells in DMEM (Sigma #D6546) supplemented with 100 U/mL penicillin, 100 µg/mL streptomycin (Gibco #15140-122) 2 mM L-glutamine, (Gibco #25030-081) and 2% FCS (Biochrom #S0115).

LASV was diluted in sterile 0.9% NaCl (BRAUN) to 10³ FFU (focus forming units) per inoculum. Inoculations were performed under isoflurane anaesthesia. For i.n. inoculation, 25 µL of inoculum were administered into one naris. Anesthetized animals were held upright for approximately 10 seconds to allow the inoculum to flow down the respiratory tract. For subcutaneous (s.c.) inoculation 100 µL of inoculum were injected into the nuchal fold. Intraperitoneal (i.p.) inoculation used 200 µL of inoculum.

Analysed endpoints for i.p., i.n., or s.c. inoculated animals were 3, 7, 14 and 28 days post-infection (dpi). In this study, 80 animals were used in groups of 6 (3 female and 3 male), with the exception of the s.c. 3 dpi-group, which was restricted to only 3 males due to insufficient animal supply.

For the i.p. and s.c. routes three control animals were mock-infected and six control animals for i.n. route to be euthanized on the same days as the LASV-infected animals.

### Sampling and blood analytes

Sampling was performed at predetermined endpoints. Urine was extracted directly from bladders using a syringe, when possible. Lumens of empty bladders were sampled with pre-wetted swabs, placed in 300 µL PBS, when urine was unavailable. Pre-wetted swabs were used for oral and rectal sampling to reduce scraping of epithelia. Samples for virological and molecular biological purposes were stored at −80°C directly after collection. Blood from cardiac punctures was collected in 2.0 mL K2 EDTA tubes (BD) for automated haematology (Abaxis VetScan HM5c) or SST-II 2.5 mL tubes (BD) for serum separation and clinical chemistry (FUJI DRI-CHEM NX500i).

### Virus isolation

Urine or medium from swabs were used for virus isolation on ATCC Vero E6 (ECACC General Cell Collection) cells in T25 culture flasks. Urine (up to 50 µL) or swab medium (up to 100 µL) were pipetted onto monolayers (80–90% confluency) with ∼500 µL of remaining culture media. The oral and rectal swabs were rinsed by pulse-vortexing with 100 µL of cell culture medium. The virus isolation flasks were incubated for 1 h at 37°C, 5% CO_2_. Afterwards, 5 mL of culture medium were added and incubated for 7 days. 140 µL of supernatant were sampled on 0, 5, (10) & 14 dpi for bladder swab/urine or 0, 7, 14 dpi for oral and rectal swabs and inactivated in AVL-buffer for RT–PCR quantification. Cell culture flasks were split at 7 dpi 1:2 into two T25 flasks, transferring the original supernatant to the flasks and adding 2.5 mL fresh medium. The cells were cultured 7 additional days (totalling 14 days). Isolations remaining RT–PCR-negative or with increasing Cq-values were designated non-infectious. RT–PCR quantification was performed also on the original sample, if sufficient volume was available.

### RNA extraction and RT–qPCR

Tissue samples were placed directly in RLT-buffer (QIAGEN) + 1% β-mercaptoethanol and stored at −80°C until processing. Samples were weighed (rounded to 0.1 mg), then homogenized using steel beads for 10 min at 30 Hz and diluted to ≥30 mg tissue input per extraction using the RNeasy Mini extraction kit (QIAGEN) according to manufacturer’s instructions. RNAs were eluted in 50 µL nuclease-free H_2_O. Quantitative RT–PCR for LASV detection was performed using the AgPath-ID™ One-Step RT–PCR Kit with 3 µL or 5 µL RNA input. The forward primer 5’-AGG TTC ACG GAA GAA GTG TAT G-3’ (0.4 µM final concentration), reverse primer 5’-CTG GAT GGA CAT TGA AGG AAG A-3’ (0.4 µM final concentration) and a detection probe 5’–6-Fam-TGC CCT CTA TCA ACC AAG TTC AGG C-BHQ-1-3’ (0.12 µM final concentration) (IDT / TIB Mol Bio) was used. Thermocycling conditions were:

10 minutes/45°C, 10 minutes/95°C and 45 amplification cycles consisting of 15 seconds/94°C and 45 seconds/60°C. Reactions were performed on C1000 Touch (Biorad) cyclers and analysed using CFX96 CFX Maestro Version 4.1.2433.1219. Cq-value conversion to Focus Forming Unit equivalents (FFU eq.) was calculated by qRT-PCR of serially diluted extracts of a quantified infectious LASV stock.

### Histology

Tissues were fixed in 10% formaldehyde in PBS for 1–2 weeks at 4°C and rebuffered in PBS for 1–2 days before paraffin-embedding and histological preparation performed at the Core Facility of the Mouse Pathology at the University Medical Center Hamburg Eppendorf, Hamburg, Germany. Tissues samples were cut transversely (2 µm), deparaffinized, and H&E staining was performed according to standard procedures.

### Data analysis and statistics

Data were plotted and analysed using Graphpad Prism 9.1.0. For serum analytes, statistical outliers of each group were identified using Robust Regression and Outlier removal (ROUT) with a *Q*-coefficient of 0.1%. Measurements below the limit of detection were imputed using $l.o.d./\sqrt{2}$ as a set value.

One male of the i.p. 28 dpi-group was euthanized prematurely at 9 dpi due to an inflamed tail wound and excluded from analyses. Mock-infection groups of different infection routes were consolidated *post-hoc* into one control group, since no mock intergroup differences were measured.

Inoculation routes were compared against the pooled mock-infected group using Kruskal–Wallis tests with *Dunn’s multiple comparisons* and *p*-values multiplicity-adjusted, accordingly. *Person* correlation coefficients (*r*) of organ LASV-RNA burden was calculated against LASV-RNA in oral, rectal swabs, and urine respectively.

Heat maps were generated using ggplot2 (R Studio) and the colour grading was manually transferred into anatomical schematics using Paint.NET v4.1.

## Results

### LASV infection is subclinical in Natal multimammate mice

To temporally investigate the effects of LASV infection of *M. natalensis*, animals were inoculated with 10³ FFU of LASV-Josiah as a small inoculation dose with high reliability in other LF models [33,34]. Hypothesizing that disparate primary inoculation routes could influence the course of infection, virus dissemination and/or shedding in the natural reservoir, i.p.-inoculation, as a classical laboratory inoculation route, was compared to i.n. and s.c. inoculation routes, grounded on their potential importance in putative intrapopulation transmission by grooming and cohabitation or territorial fights. Natal multimammate mice, scored daily, showed no clinical or behavioural disease signs, and weight gained by LASV-infected animals was consistent with that of mock-infected animals.

**Figure 1.**
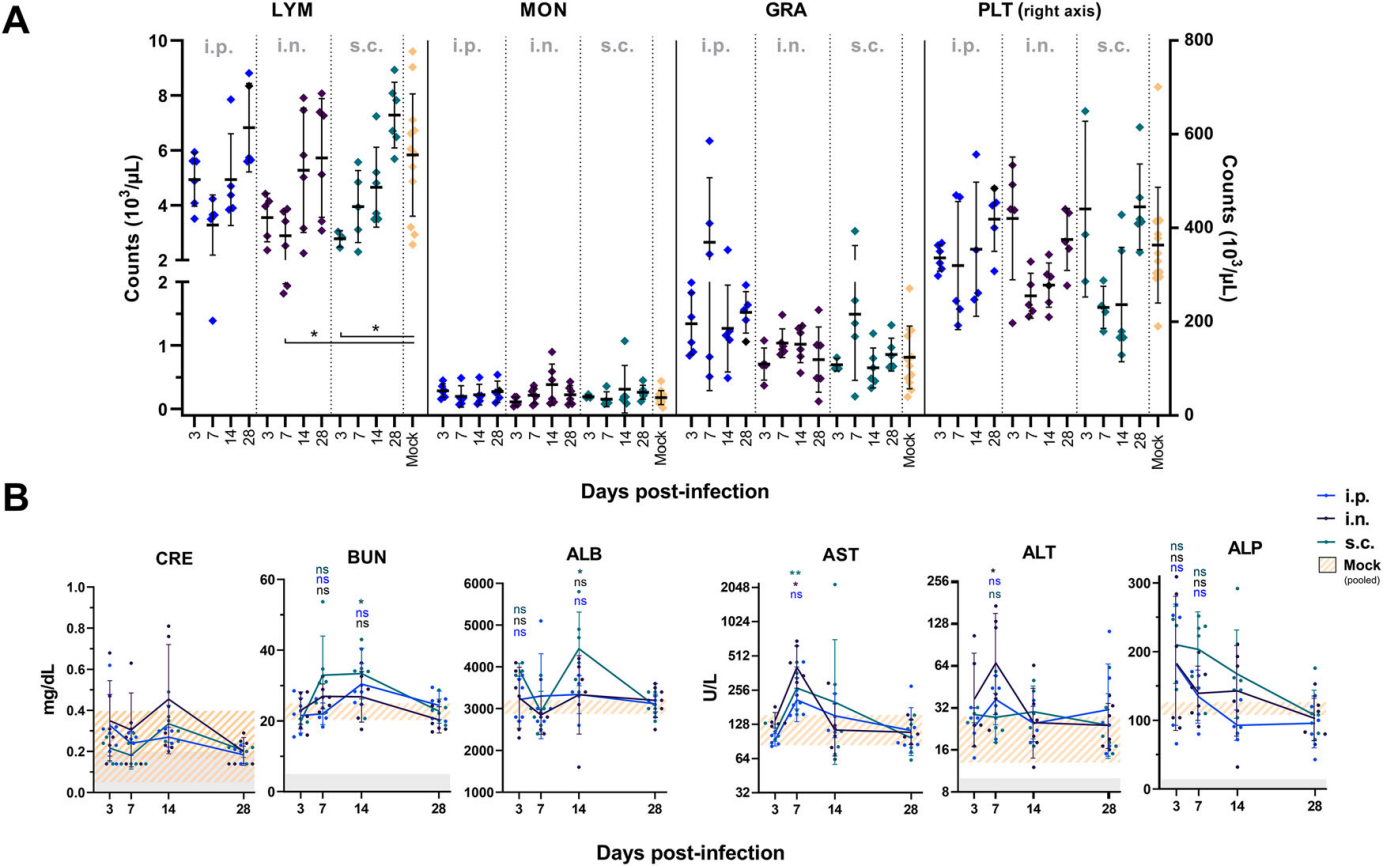
Reduced circulating lymphocytes and altered clinical serum chemistry of Natal multimammate mice after LASV infection. (A) Lymphocyte (LYM), monocytes (MON), granulocyte (GRA) and platelet (PLT) concentrations were measured from whole blood samples. Results are displayed as means (bar)±SD and individual biological replicates (*n* = 3–6). The mock-infected group pools mock-infected animals of different infection routes. (B) Sera of i.n.-, s.c.- and i.p.-inoculated animals were sampled at 3, 7, 14, and 28 dpi. Data presented for CRE, BUN, ALB and ALP as mean (line) ±SD; for AST and ALT as geometric mean (line) ±SD, biological replicates (*n* = 3–6), pooled mock mean ±SD (orange striped area); detection limit (grey area). Statistical differences of LASV-inoculations against pooled mock-infected group are indicated by *p < 0.05, **p < 0.01 and ns (non-significant), determined by *Dunn’s multiple comparisons*.

Haematological parameters were similar to our previously measured *M. natalensis* values [29]. However, an infection route-independent pattern of decreased lymphocyte counts emerged in the first week after i.n. and s.c. inoculation (*p *< 0.05), rebounding at 14 and 28 dpi (Figure 1(A)). While the pattern in i.p.-inoculated animals was similar, due to high intra-group variability, these observations were not statistically significant. Likewise, but statistically non-significant, a pattern of decreased platelets was observed in most infected animals at 7 and 14 dpi, but remained within *M. natalensis* reference ranges. No changes were discernible in monocyte or granulocyte populations.

The sera collected at euthanasia revealed a significant transient increase of mean AST concentrations during acute infection (7 dpi) of i.n.-inoculated (*p *< 0.01) and s.c.-inoculated (*p *< 0.05) animals (Figure 1(B)) compared to the pooled mock-infected group. Increases in i.p.-inoculated animals did not significantly differ from mock-infected animals. Geometric mean AST concentrations peaked to 406.6 U/L ×/÷1.535 GSD (geometric standard deviation) in i.n.-LASV-infected animals at 7 dpi. Similarly, the geometric mean ALT concentrations were elevated (*p *< 0.05) in i.n.-inoculated animals at 7 dpi (66.6 U/L ×/÷2.313 GSD) compared to mock-infected animals. Mean serum ALP concentrations were increased at 3 dpi for all groups with up to 247 U/L detected in sera, although this was statistically not significant (*p* = 0.25 (i.n.), 0.15 (i.p.), 0.10 (s.c.)), compared to normal values of mock-inoculated animals. Mean ALP concentrations for i.n.- and i.p.-inoculated groups returned to baseline by 7 dpi, while ALP concentrations seemed to remain elevated in s.c.-inoculated animals at 7 dpi. Throughout the infection, ALP values of all groups returned to levels of mock-inoculated animals. BUN and CRE, markers often elevated in severe LF cases, expectedly, were not elevated in the Natal multimammate mice, with the exception of BUN in s.c.-inoculated animals 14 dpi (*p *< 0.05). Of note, at 14 dpi CRE measurements of all LASV-inoculated animals were above the lower limit of detection, which was previously not the case, still no statistical significance was observed. During the study, measurements of GGT were performed as another indicator of liver health, but were generally below the detection limit throughout infection (not shown), making GGT unfit as an analyte during LASV infection for *M. natalensis*.

### LASV replicates systemically in Natal multimammate mice and tissue viral load dynamics are disparate between inoculation routes

**Figure 2.**
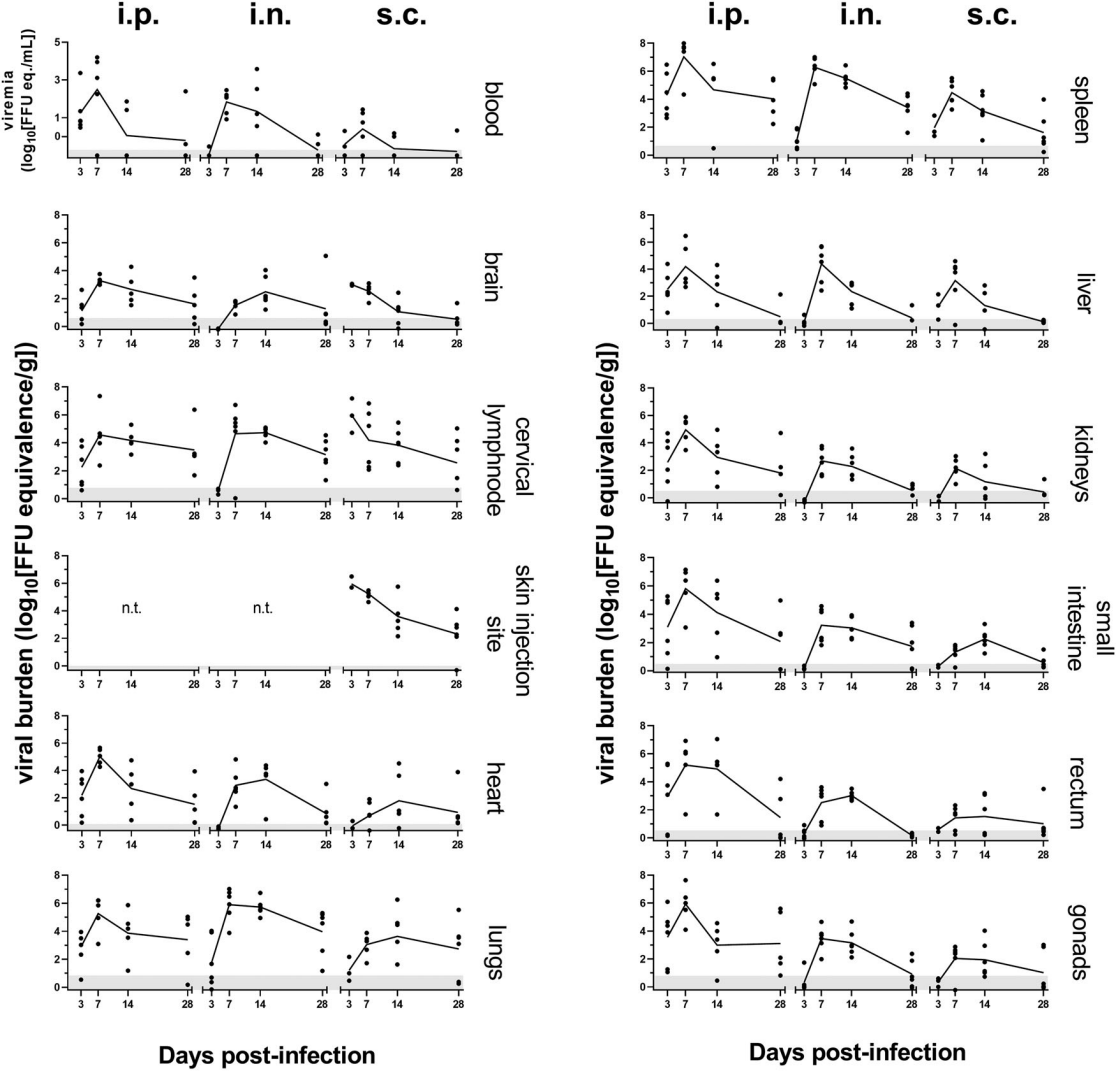
Route of inoculation influences LASV replication dynamics culminating in systemic infection of *M. natalensis*. Quantification of LASV-RNA loads for i.p.-, i.n.- and s.c.-inoculation at 3, 7, 14, and 28 dpi in all analysed organs. Skin of the inoculation site was not tested (n.t.) for i.n. & i.p. inoculations. Graphs present geometric means (line) and biological replicates (dot) (*n* = 3–6), detection limit (grey area).

We sought to determine the susceptibility of our outbred *M. natalensis* colony to LASV, and to assess whether the inoculation route affects tissue distribution and viral loads. We examined LASV-RNA loads over time in blood and target tissues that are classically involved in other rodent and NHP LASV infection models (Figure 2) [34]. No sex-specific viral RNA load differences were observed. I.p. inoculation resulted in rapid dissemination of LASV to many organs, with RNA detected by 3 dpi, averaging 10^3^ FFU eq./g in lungs and 10^4^ FFU eq./g in spleens. Accumulation of LASV-RNA in i.p.-inoculated animals reached maximal levels in the lungs, spleen and blood at 7 dpi, decreasing slightly by 14 dpi, yet remained detectible at 28 dpi. Viral RNA loads in tissues had a markedly different kinetic in both the i.n.- and s.c.-inoculation routes, compared to i.p. inoculation and compared to each other. In i.n.-inoculated animals, little-to-no LASV-RNA was detected in the organs at 3 dpi. By 7 dpi, however, viral accumulation of approximately 10^6^ FFU eq./g in lungs and spleens was measured on average, similar as for i.p. inoculation. S.c.-inoculation generally resulted in lower organ LASV-RNA loads (lung 7 dpi: i.n. vs s.c., *p *< 0.05; spleen 7 dpi: i.p. vs s.c., *p *< 0.05; *Kruskal–Wallis tests with Dunn’s multiple comparisons*), between 10^2^ and 10^5^ FFU eq./g in these organs at 7 dpi. Temporal patterns of LASV-RNA loads in organs were reflected in the blood of the animals of respective inoculation routes, but with titres lower by approximately a factor of 10,000. While the majority of animals at 28 dpi were not viremic, LASV-RNA clearance from spleens and lungs was delayed, independent of the inoculation route.

**Figure 3.**
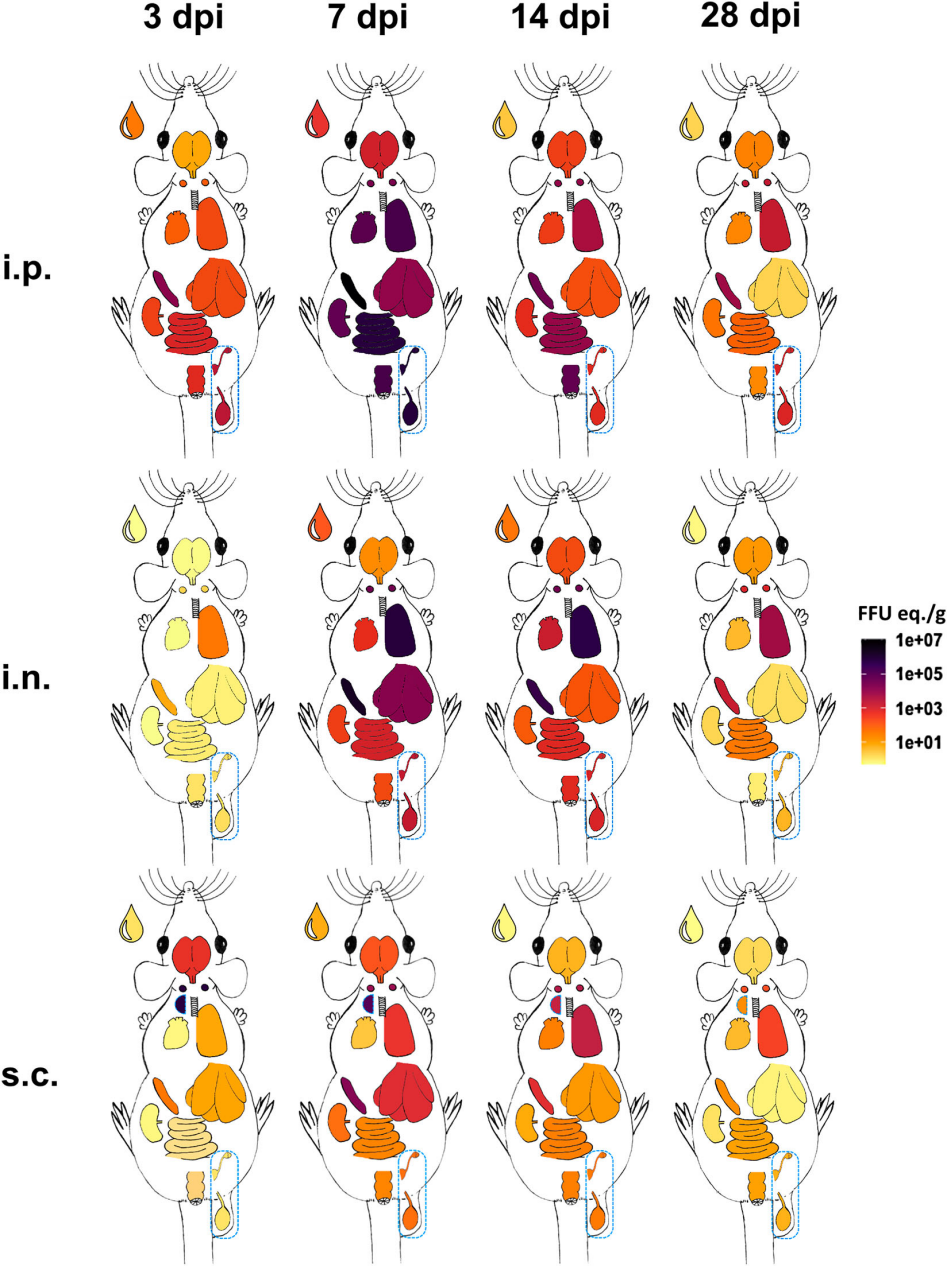
Disparate inoculation routes result in distinct differences in LASV dissemination in *M. natalensis*. Examined organs following i.p., i.n. or s.c. inoculation are displayed as outlines (cranial to caudal): blood (drop), brain, cervical lymph nodes, skin at injection site (half circle; s.c. only), lungs, heart, liver, spleen, kidneys, small intestine, gonads (ovaries or testes), rectum. Geometric means of LASV-RNA concentrations in organs at 3, 7, 14 and 28 dpi are spectrally heat-mapped. Light yellow: detection limit/RT-qPCR-negative; dark purple: 10^7^ FFU eq./g.

Independent of the inoculation route, exposure to LASV resulted in systemic infections in the Natal multimammate mice*,* evidenced by the presence of LASV-RNA in all sampled organs, far exceeding input RNA levels (Figure 2). Plotting the geometric mean LASV-RNA loads for sampled organs spectrally in an anatomical heat-map (Figure 3) allows comprehensive visualization of the differences in viral RNA loads based on infection route and time. I.p.-inoculation resulted in early viral RNA detection in all abdominal organs, as expected, progressing to high viral loads in the lungs and hearts by 7 dpi. In contrast, in i.n.-inoculated animals, a low abundance of LASV-RNA was measured in lungs and spleens by 3 dpi, spreading fulminantly to all tested organs by 7 dpi. The kinetics in animals inoculated s.c. also differed. Early and strong local replication was observed at the skin injection site (Figure 3; s.c. half circle) with immediate spread to cervical lymph nodes and detection of LASV-RNA in brains at 3 dpi. In comparison, even in the systemic infection following i.p.-inoculation, LASV-RNA was only detected in some of the animals’ brains after 3 dpi, while the cerebral involvement in i.n.-inoculated Natal multimammate mice was first observed at 7 dpi. Additional disparate patterns between inoculations routes emerged by 3 dpi, evolving to peak organ burden and pattern differences at 7 dpi, such as increased viral RNA burden after s.c.-inoculation in spleens and livers, compared to i.n.-inoculation at 3 dpi, which on the contrary, did not lead to higher titres in animals at 7 dpi, but lower titres than in i.n.-inoculated animals.

Secondary lymphoid tissues (lymph nodes and spleens) were especially supportive of viral replication, independent of the inoculation route, while lungs and livers were secondary LASV hot-spots, even by inoculation routes not directly targeting these organs. However, viral loads in these secondary hot-spots were distinct, especially in s.c.-inoculated animals.

In i.p.-inoculated animals, organs involved in excretion, residing in the abdominal cavity (kidneys, small intestine and rectum), displayed, expectedly, LASV-RNA by 3 dpi, while the same organs had detectible LASV-RNA in i.n.- or s.c.-inoculated animals beginning at 7 dpi. The LASV-RNA titres in the gonads, independent of sex, followed excretion organ values within respective inoculation groups. I.p.-inoculated animals uniquely had reduced clearance of LASV-RNA from the gonads.

Based on the experiment’s temporal resolution, peak viral burden was at 7 dpi for all inoculation routes, after which LASV-RNA levels decreased. An exception was seen in the brains of i.n.-inoculated animals, where the measured mean LASV-RNA loads peaked at 14 dpi. Nevertheless, all inoculation routes were delayed in the clearance of LASV-RNA from lungs and lymphoid tissues up to the final 28 dpi time point.

### LASV shedding by Natal multimammate mice is inoculation route-dependent

**Figure 4.**
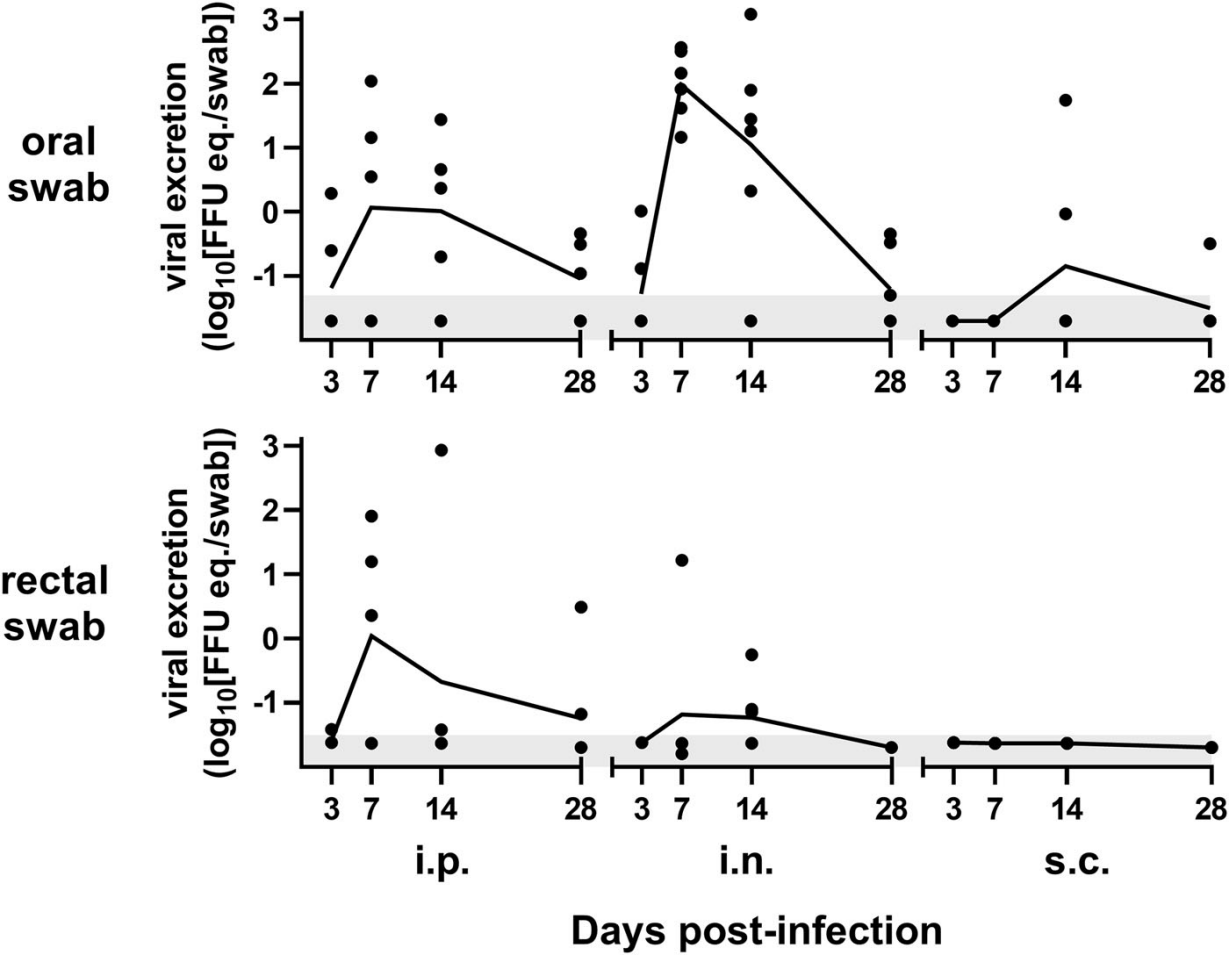
LASV-RNA is shed orally and rectally in *M. natalensis*. RT-qPCR of LASV-RNA from oral or rectal swabs from *M. natalensis*, inoculated either i.p., i.n. or s.c. and sampled at 3, 7, 14, and 28 dpi. Graphs present geometric means (line) and replicate (*n* = 3–6).

Transmission is necessary for viral maintenance in reservoir populations. To assess whether various inoculation routes result in differential shedding of LASV in *M. natalensis*, oral and rectal swabs and urine of the animals were analysed for LASV-RNA and viable virus. Over the course of infection, LASV-RNA was detected at low titres (up to 10²–10³ FFU eq.) in oral and rectal swabs from several animals (Figure 4). Oral swab LASV titres correlated most closely with the virus burden in lungs (*r* = 0.70) spleens (*r* = 0.66), and livers (*r* = 0.62), while rectal shedding correlated most closely with skin (*r* = 0.69) (only sampled for s.c.-inoculation), rectum (*r* = 0.51) and reproductive organs (*r* = 0.47). Interestingly, oral or rectal RNA-shedding was not correlated with viremia levels.

I.n.-inoculated animals consistently had the most LASV-RNA in oral swab samples at 7 and 14 dpi, while s.c.-inoculated animals had positive oral swabs only at 14 dpi. Viral RNA loads in oral swabs, when present, were of similar magnitude when compared between i.n. and i.p.-inoculated animals. Only few rectal swabs were positive for LASV from animals inoculated i.n. or i.p., while no rectal swabs of s.c.-inoculated Natal multimammate mice tested positive.

Urine is reported as a putative vehicle for LASV transmission, but published virological data on this topic is incomplete. Virus isolation attempts from RT–PCR-positive bladder swabs and urine from this study upon euthanasia were mostly unsuccessful (Table 1). Infectious virus was isolated from 4/67 (∼6%) animals. Only 33% of the animals (22/67) shed LASV-RNA at titres ranging from 10^0^ to 10^4^ FFU eq./mL in urine or bladder swab samples, correlating with the viral burden in kidneys (*r* = 0.73) and blood (*r* = 0.55). All successful LASV isolations originated from bladder swabs instead of urine, two from i.p.-inoculated animals at 3 dpi, the two other virus-positive bladder swabs were from animals euthanized at 7 dpi (one i.p., one i.n.). Irrespectively, virus isolation attempts from RT–PCR-positive oral and rectal swabs were all unsuccessful. Of the few successful LASV isolations from bladder swabs, corresponding lower limits of input for viral isolation were 3×10^−1^, 4×10^1^, 4×10^2^ and 1×10^3^ FFU eq., according to RT-qPCR, demonstrating no discernible cut-off since the overwhelming magnitude of isolations from urine, oral and rectal swabs in the RNA range of 3×10^1^–4×10^2^ FFU eq. were unsuccessful.

**Table 1.** Viable LASV isolation from urine and swab samples of adult *M. natalensis*. Summary of RT-qPCR testing of urine/bladder swabs, oral swabs and rectal swabs and *in vitro* virus isolation attempts from 67 animals inoculated via different routes.

Route	*M. natalensis* infected with LASV	LASV-RNA PCR positive primary samples	Positive LASV virus isolations {from swabs}
Urine/bladder swab			
i.p.	22	14	3 {3/3}
i.n.	24	6	1 {1/1}
s.c.	21	2	0
Total	67	22/67 (32.8%)	4/67 (6.0%)
Oral swab			
i.p.	22	13	0
i.n.	24	16	0
s.c.	21	3	0
Total	67	32/67 (47.8%)	0
Rectal swab			
i.p.	22	8	0
i.n.	24	4	0
s.c.	21	0	0
Total	67	12/67 (17.9%)	0

### LASV infection causes immune cell infiltration and histologic alterations in Natal multimammate mice

**Figure 5.**
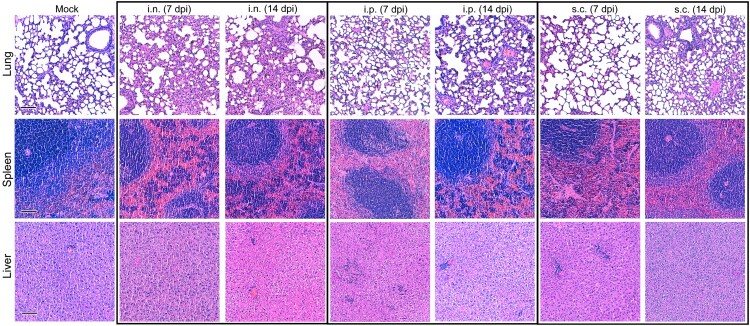
LASV infection leads to immune cell infiltration in lungs, spleen and liver of Natal multimammate mice. Histologic analyses of *M. natalensis* lungs, spleen and liver at 7 and 14 dpi of LASV- or mock-inoculated were performed. Representative images of H&E-stained tissues show morphological alterations in infected animals, including immune infiltrates in lungs and livers, interstitial thickening and disruption of the spleen.

We observed extensive LASV replication in the Natal multimammate mouse reservoir, which was subclinical with clinical analytes exhibiting only short-lived alterations (Figure 1). To investigate LASV-specific responses, we assessed selected spleen, lung and liver tissues at the time of peak viral loads at 7 dpi as well as at 14 dpi. Histological examination revealed obvious changes in the tissue’s micro-structure. Immune cell infiltration in the lung was most pronounced in i.n.-inoculated animals, disrupting alveoli structures by interstitial thickening, while lungs of s.c.-inoculated animals showed minor immune cell infiltrates 7 and 14 dpi. I.p. inoculation only affected the lung tissue at 14 dpi but not 7 dpi (Figure 5). Spleens of LASV-infected animals displayed clustered immune cell infiltrates in an otherwise eosinophilic red pulp, with the exception of i.p.-inoculated animals at 7 dpi and s.c. inoculation at 14 dpi (Table 2 and Figure 5). Immune cell infiltrates in livers were observed in LASV-infected animals, except i.n.-inoculated animals at 7 dpi and after s.c. inoculation at 14 dpi. Of note, s.c.-inoculated animals displayed similar but less pronounced changes, concomitant with their overall lower LASV burden. This histological analysis suggests elicitation of an extensive immune response during the otherwise subclinical course of LASV infection in adult Natal multimammate mice.

**Table 2.** Histological evaluation of lung, spleen and liver tissues show immune cell infiltration in *M. natalensis* infected with LASV (14 dpi). Summary report of histological findings by blinded evaluation.

Route organ		i.p.	i.n.	s.c.	Mock
Lung	7 dpi	• normal organ structure • no abnormalities	• immune cell infiltration • restricted alveoli-microstructure	• minor immune cell infiltration	• no abnormalities
	14 dpi	• immune cell infiltrates • abundant alteration of alveoli-microstructure	• immune cell infiltrates • extensive alterations of alveoli-microstructure	• minor immune cell infiltration • normal organ structure	
Spleen	7 dpi	• normal organ structure • no abnormalities	• immune cell infiltrates • alterations of the follicular structure	• immune cell infiltrates • alterations of the follicular structure	• normal organ structure • no abnormalities
	14 dpi	• immune cell infiltrates • alterations of normal follicular structure	• immune cell infiltrates • alterations of normal follicular structure	• normal organ structure • no abnormalities	
Liver	7 dpi	• abundant immune cell infiltrates	• normal organ structure • no abnormalities	• immune cell infiltrates	• normal organ structure • no abnormalities
	14 dpi	• immune cell infiltrates	• immune cell infiltrates	• normal organ structure • no abnormalities	

## Discussion

LASV is disproportionally understudied compared to viral haemorrhagic fevers that cause fewer annual morbidities and less mortality. More than 40 years ago, *M. natalensis* was identified as a primary natural reservoir of LASV, along with other Muridae species [35,36], yet we have a limited understanding of general virus-host interactions or virus dynamics within LASV reservoir species.

Herein, we develop a contemporary LASV infection system for this natural reservoir using a genetically heterogenous outbred breeding colony to investigate the dissemination of LASV in adult *M. natalensis* [14]. Acute infection in young adult animals was investigated, as they make up the bulk of the *M. natalensis* population encountered by humans [37]. We show Natal multimammate mice develop subclinical disease, widespread viral dissemination in the organs, and demonstrate LASV-induced histologic changes. Furthermore, we show that the route of inoculation results in disparate infection dynamics and shedding.

The primary inoculation sites of the i.p. and s.c. routes were regions of early LASV-RNA accumulation, exceeding the inoculum of 10³ FFU in each animal, indicating local replication. Yet, deep lung tissue of i.n.-inoculated animals did not show this early accumulation, since early replication likely occurs in the upper respiratory tract, which was not sampled in this study. I.p. inoculation, analogous to intravenous administration, is a classical route for disease modelling to facilitate rapid and systemic virus spread and replication, often resulting in pathogenesis [14], making it a standard inoculation route in vaccine and antiviral research. Accordingly, i.p. inoculation with LASV in Natal multimammate mice led to early systemic viral replication and high LASV loads. Despite the extensive LASV replication following i.p. inoculation, which does not occur naturally, LASV still did not potentiate clinical disease, likely due to a tightly co-evolved virus-host relationship, demonstrating the inherent resistance of *M. natalensis* against LASV-induced disease [14,28,34]. We utilized i.p. inoculation to contrast whether naturally relevant inoculation routes might lead to differences in LASV replication dynamics, organ tropism, shedding and/or pathologic responses. I.n. inoculation resulted in low initial LASV replication, localized to lungs and spleens, yet ultimately resulted in systemic LASV organ burden, similar to early i.p.-inoculation loads. The inoculation routes also differed in the clearance of LASV in *M. natalensis*. I.n.-inoculated animals resolved infection with delayed clearance from lungs, spleens and the cLNs, while i.p.-inoculated animals struggled to clear the viral RNA from a multitude tissues as late as 28 dpi. No specific sex-dependent differences were observed; however, more study subjects likely are required to reliably identify such effects.

Peak LASV-RNA titres in organs of s.c.-inoculated *M. natalensis* were lower than for either i.p. or i.n. inoculation, except for early brain and cLN levels, but viral clearance was similar to that for i.n. inoculation. Only i.p. inoculation of *M. natalensis* is published, however, the LASV-related arenaviral lymphocytic choriomeningitis virus (LCMV) also readily accumulates in lymph nodes after s.c. inoculation of its natural reservoir, *Mus musculus*, but with less splenic involvement when compared to intravenous LCMV inoculation [38].

These differences in viral loads and dynamics highlight that a consideration for inoculation route is crucial for virological or immunological questions that remain to be addressed in *M. natalensis* regarding LASV infection. Differential inoculation routes likely lead to disparate initial immune cells targets. Entry via the mucosa during i.n. inoculation involves lung epithelia and alveolar macrophages, which have evolved to facilitate immune responses against invading pathogens. I.p. inoculation circumvents this and might also target infection of immature peritoneal monocytes, for which the potent type I interferon antagonism of LASV [39–41] might lead to mal-adaptive immune responses.

Similar to i.p., i.n. inoculation of Natal multimammate mice consistently caused a systemic infection, despite the necessity of LASV to transverse evolutionary-adapted immune barriers of the respiratory tract. This route likely mimics a natural mode of inter-rodent transmission in the wild, as infectious excretions of *M. natalensis* can potentially easily be transmitted onto the mucosa of naïve adult animals within a colony. We observed oral shedding of LASV RNA in adult *M. natalensis* after infection, however, intradermal or s.c. inoculation of animals by biting is less likely to be a route of transmission within populations, since the required intraspecies aggression is less common in *M. natalensis* [28]. Additionally, the viral loads measured in oral swabs after s.c. inoculation were diminutive, similar to loads found in saliva of *M. natalensis* after i.p. Morogoro virus inoculation [24].

We hypothesize that nasal mucosal exposure could be an important entry site in nature. Oral swabs of i.n.-inoculated animals displayed high viral RNA loads similar to i.p. inoculation. Interestingly, despite the vicinity of the s.c. inoculation in the nuchal fold to salivary glands, s.c. inoculation resulted in relatively little oral viral RNA shedding late during the infection. Rectal LASV RNA shedding was observed for i.n.-inoculated animals, while s.c.-inoculated animals consistently tested negative, again showing important differences between inoculation routes, with i.n. inoculation allowing for potential virus transmission, whereas s.c. inoculation would be unlikely to allow for transmission, due to the low or absence of LASV shedding.

Virus isolation attempts from urine of adult *M. natalensis* were consistently negative. While urinary bladder swabs only made up 17.9% of our virus isolation attempts, they comprised all of the successful virus isolations. LASV isolation from urine of adult *M. natalensis* was previously demonstrated in one report from a single low-titre urine sample late in infection of an adult animal [14], while comprehensive reviews of McCormick and Fisher-Hoch cite unpublished data of lifelong highly viruric animals after *in utero* or postpartum infection [42,43]. These publications, together with our findings, highlight remaining questions on whether infectious LASV virions are excreted in sufficient quantities in urine from *M. natalensis*, infected as adults, and stress the need for comprehensive studies on this topic. The age of the animals at the time of infection should be emphasized. It has been repeatedly shown that *M. natalensis* and other rodents that are infected with arenaviruses *in utero*, perinatally or in neonatal stages postpartum, remain persistently infected and shed virus in urine for long periods [23–26], which also holds true for LASV, as preliminary studies demonstrated [14]. Likewise, heterologous LASV infection in neonatal *M. musculus* resulted in prolonged shedding in the urine [44]. Therefore, persistent infection and shedding after vertical transmission or early postpartum infection is a plausible mode of maintenance of LASV in wild populations of *M. natalensis* and remains a potential source of rodent-to-human transmission. For the LASV-related Morogoro virus, horizontal transmission to neonates of *M. natalensis* from persistently infected individuals reduced the chance of persistent infection with this LASV-related arenavirus by 20–60% compared to s.c.-inoculation at day two after birth [23]. Likewise, the inoculation route might be a major factor in LASV persistence and needs to be thoroughly considered if experimental data are to be applied to virus transmission in a wild population. Natal multimammate mice infected as adults, which are able to immunologically react to LASV infection and do not seem to efficiently shed infectious virions as demonstrated herein, likely play a dispensable role in the maintenance of the virus in the population and transmission to humans.

LASV infection of *M. natalensis* has been predominantly described as low to non-pathogenic due to co-evolutionary virus-host adaptations. While wild-caught individuals of *M. natalensis* infected with LASV displayed higher ratios of pathophysiological changes compared to LASV-free animals [45], helminth and protozoan co-infections of these animals confounded interpretations. In more recent studies, no effects of LASV on growth could be detected in wild populations [46]. Our experimental infections with 10³ FFU of LASV partially confirmed prior histopathological results, where serum chemistry analytes, as well as histological examinations, showed minor pathological changes in young adult *M. natalensis*, despite no significant influences of LASV infection on the clinical status, such as body weight changes or signs of disease. Since the experiments were conducted with the well-studied Josiah strain of LASV (a human isolate) with passaging on Vero E6 cells, an attenuation of the virus in a Natal multimammate mouse host due to a prior primate adaptation cannot be fully excluded. However, recent preliminary infection experiments with a LASV strain isolated from a Natal multimammate mouse also showed no alteration of the clinical status of the animals [28]. Additional infection experiments with reservoir isolates of LASV, such as the Soromba-R isolate, or inoculation with homogenates of LASV-infected Natal multimammate mice without passaging will help to classify the true extent of the natural host responses.

The pathological changes seen in the histological examination of LASV-infected *M. natalensis* in this study show similarity to the infection course of its arenaviral cousin LCMV in its reservoir host, which induces transient disease following infection, and is cleared within 7–10 days [47] (dependent on infectious dose and strain), but is also subclinical in mice infected *in utero* [48]. Dependent on the inoculation route, the extent of histological change in *M. natalensis* differed. S.c. inoculation resulted in less pronounced histological changes, while i.p. and i.n. inoculations of LASV displayed more widespread alterations. Future studies of additional organs and time points and staining targets will provide a detailed characterization of potential tissue pathology and resolution.

I.p., s.c. and aerosol inoculation with LASV in other rodent *in vivo* models, such as mice and guinea pigs, established as lethal pathology models, results in histological changes of a similar type as the infected Natal multimammate mice in this study; with interstitial pneumonia, eosinophilic red pulp of the spleen and immune infiltrations and diffuse apoptosis in the liver, as well as serum AST and ALT peaks between 7 and 9 dpi [33,49–51]. However, the extent of the observed alterations in the otherwise subclinical Natal multimammate mouse infection was much lower compared to the pathology models. Additionally, the only study comparing inoculation routes in a lethal Strain 13 guinea pig pathology model for LF did not observe the major difference between s.c. and aerosol inoculation [49], further demonstrating the scientific uniqueness of natural reservoir hosts as study subjects.

The increasing emergence and re-emergence of zoonoses highlight the need for an understanding of how zoonotic viruses interact with their animal reservoirs. We, therefore, present a detailed examination of the course of LASV infection in adults of *M. natalensis*, showing a systemic subclinical infection with histopathologic immune-related responses. Furthermore, we discovered that the inoculation route influences the clinical parameters, viral dissemination in the organs and shedding of viral RNA, leading to important implications for the study of natural infection and transmission within *M. natalensis* populations and to humans.

## Data Availability

The data that support the findings of this study are available from the corresponding author upon reasonable request.
